# Macrophage-targeted anti-CCL2 immunotherapy enhances tumor sensitivity to 5-fluorouracil in a Balb/c-CT26 murine colon carcinoma model measured using diffuse reflectance spectroscopy

**DOI:** 10.1186/s12865-022-00493-5

**Published:** 2022-04-23

**Authors:** Shelby N. Bess, Gage J. Greening, Narasimhan Rajaram, Timothy J. Muldoon

**Affiliations:** Department of Biomedical Engineering, University of Arkansas, 1 University of Arkansas, Fayetteville, AR 72701 USA

**Keywords:** Colorectal cancer, Tumor-associated macrophages, Diffuse reflectance spectroscopy, Immunomodulation

## Abstract

**Background:**

Immunotherapy in colorectal cancer (CRC) regulates specific immune checkpoints and, when used in combination with chemotherapy, can improve patient prognosis. One specific immune checkpoint is the recruitment of circulating monocytes that differentiate into tumor-associated macrophages (TAMs) and promote tumor angiogenesis. Changes in vascularization can be non-invasively assessed via diffuse reflectance spectroscopy using hemoglobin concentrations and oxygenation in a localized tumor volume. In this study, we examine whether blockade of monocyte recruitment via CCL2 (macrophage chemoattractant protein-1) leads to enhanced sensitivity of 5-fluorouracil (5-FU) in a CT26-Balb/c mouse model of CRC. It was hypothesized that the blockade of TAMs will alter tumor perfusion, increasing chemotherapy response. A subcutaneous tumor model using Balb/c mice injected with CT26 colon carcinoma cells received either a saline or isotype control, anti-CCL2, 5-FU, or a combination of anti-CCL2 and 5-FU.

**Results:**

Findings show that 12 days post-treatment, monocyte recruitment was significantly reduced by approximately 61% in the combination group. This shows that the addition of anti-CCL2 to 5-FU slowed the fold-change (change from the original measurement to the final measurement) in tumor volume from Day 0 to Day 12 (~ 5 fold). Modest improvements in oxygen saturation (~ 30%) were observed in the combination group.

**Conclusion:**

The findings in this work suggest that the blockade of CCL2 is sufficient in the reduction of TAMs that are recruited into the tumor microenvironment and has the ability to modestly alter tumor perfusion during early-tumor response to treatment even though the overall benefit is relatively modest.

**Supplementary Information:**

The online version contains supplementary material available at 10.1186/s12865-022-00493-5.

## Introduction

Colorectal cancer (CRC) is estimated to account for 149,500 new cancer cases annually in the United States, making it the 4^th^ most common cancer type overall (behind breast, prostate, and lung), and resulting in 52,980 annual deaths [1]. Locally-advanced CRC (stages II-III), which accounts for approximately 20% of cases, describes cancer that has spread from the site of the primary tumor to surrounding tissue or lymph nodes but has not metastasized [2–4]. The standard of treatment for locally-advanced CRC is neoadjuvant chemoradiotherapy (CRT) using 5-fluorouracil (5-FU), followed by total mesorectal excision (TME) surgery [5]. Following neoadjuvant CRT, biopsies are examined to determine pathologic response. Ideally, patients will exhibit pathologic complete response (pCR) when there is an absence of residual cancer cells in a histological examination. Patients achieving pCR have a reduced risk of distal recurrence [2]. However, pCR is achieved in less than 30% of cases, resulting in distal recurrence rates of 25%, which is the primary cause of CRC-related death [2]. Thus, the development of more effective treatments for CRC is in urgent need. An emerging strategy called immunomodulation therapy, or immunotherapy, has gained clinical momentum in recent years to aid neoadjuvant CRT in reducing tumor burden and recurrence risk [6–8].

Immunotherapy in CRC is a broad approach aimed at modulating the immune system to inhibit checkpoints of pro-tumor pathways to increase tumor sensitivity to chemotherapy [6]. One specific immunotherapy strategy is the blockade of CCL2/MCP-1 (monocyte chemoattractant protein-1), an elevated cytokine during CRC progression which recruits monocytes to the tumor microenvironment [9]. Monocytes differentiate into tumor-associated macrophages (TAMs), which have pro-tumor functions in CRC [10–19], although conflicting studies have reported anti-tumor functions of TAMs at tumor margins [20–23]. Pro-tumor functions of TAMs include direct secretion of angiogenic growth factors (GFs) leading to an increase in vascular endothelial growth factor (VEGF) [10, 19, 24] and ECM-degrading matrix metalloproteases (MMPs) which allow for the release of ECM-sequestered angiogenic GFs [10, 17]. FDA approval of anti-VEGFs, such as bevacizumab, has shown significant benefit in overall survival in breast cancer; however, it has shown no significant benefit in terms of overall survival, leading to many clinicians restricting its use and a shift in focus to modulation of cytokines [25]. CCL2-mediated TAM infiltration is linked to increased inflammation, angiogenesis, and tumorigenesis; therefore, anti-CCL2 immunotherapy has the potential to reduce tumor burden and recurrence risk [12, 26].

Recent research has explored CCL2 blockade as an immunotherapy strategy in various mouse models. Popivanova et al. showed that CCL2 blockade reduced neovascularization and colon tumor size in Balb/c mice [27]. Zhu et al. showed that administration of anti-CCL2 in combination with temozolomide chemotherapy in C57BL/6 mice with GL261 glioma significantly prolonged survival [28]. Svensson et al. demonstrated that CCL2 blockade in FVB/N mice with MCF-7 breast cancer decreased TAM infiltration and reduced estrogen-stimulated cancer growth [29]. Kirk et al. showed that delivering anti-CCL2 in combination with docetaxel chemotherapy in SCID mice with C4-2B prostate adenocarcinoma inhibited tumor progression [30].

It has been shown that TAMs not only have a role in tumor progression, but also in cancer chemoresistance. Zhang et al. demonstrated that TAMs directly contribute to 5-FU chemoresistance in CRC and concluded that TAM pathways (such as CCL2) were potential immunotherapy targets to increase the efficacy of 5-FU chemotherapy [13]. Although studies have investigated the use of a CCL2 antagonist on the recruitment of macrophages and their interactions between other immune cells, no studies have correlated macrophage recruitment to the functional changes in the tumor microenvironment (i.e., hypoxia).

This study further examines the role of the CCL2/MCP-1 axis in colorectal cancer via antibody mediated blockade of CCL2 combined with 5-FU chemotherapy in Balb/c mice with subcutaneous CT26 colon carcinoma allografts. Mice were separated into five groups: saline control, isotype control. anti-CCL2 immunotherapy, 5-FU chemotherapy, or combination therapy groups. 5-FU was expected to alter tumor hemodynamics due to a rapid decrease in cellular metabolism leading to a decrease in oxyhemoglobin conversion to deoxyhemoglobin. The addition of anti-CCL2 was expected to modulate this effect. Through the duration of the study, tumor perfusion in vivo was longitudinally measured via DRS to quantify perfusion metrics such as tissue hemoglobin content (THC) and tissue oxygen saturation (StO_2_). DRS measurements were correlated to end-point immunohistochemistry (IHC) markers of hypoxia (carbonic anhydrase-9) and microvasculature (CD31), along with tumor-associated macrophage recruitment (CD68) and M2 tumor-associated macrophage recruitment (CD163). This study examines one major hypothesis: CCL2 blockade in the tumor microenvironment increases the sensitivity of CT26 tumors to 5-FU chemotherapy, which is quantified via longitudinal tumor growth, DRS-derived metrics and IHC analysis.

## Materials and methods

### Cell line

CT26 (ATCC®, CRL-2638™) cells were maintained under standard conditions in Roswell Park Memorial Institute (RPMI)-1640 medium (ATCC®, 30–2001™) supplemented with 10% fetal bovine serum (ATCC®, 30–2020), 1% antibiotic antimycotic solution (Sigma-Aldrich, A5955-100ML), and 0.2% amphotericin B/gentamicin (Thermo Fisher Scientific, R015010). CT26 cells were brought to the third passage for a total of approximately 5 × 10^6^ cells prior to implantation.

### Animal model

All animal experiments were approved by the University of Arkansas Institutional Animal Care and Use Committee (IACUC #18060 and #21014) and carried out in accordance with the National Institutes of Health Guide for the Care and Use of Laboratory Animals and the ARRIVE guidelines. All efforts were made to minimize animal suffering and to reduce the number of mice used. Nine-week-old female Balb/c mice (n = 95) were obtained from The Jackson Laboratory (Bar Harbor, ME, USA). Upon arrival, mice were housed in groups of three at 23 °C ± 1 °C and 50% ± 10% humidity with a 12:12-h light–dark cycle and had access to water and standard rodent food ad libitum. Mice underwent one week of environmental acclimation, including daily handling (1 min per mouse) for stress adaptation to future handling during measurements. Five days after arrival, hair on the injection site (left flank) was removed via shaving and a depilatory lotion, and then cleaned. Seven days after arrival, the mice underwent subcutaneous (SQ) injection of 1 × 10^6^ CT26 cells into the left flank. Tumors were allowed to grow until they reached 75 ± 5 mm^3^ (day 0), as measured via ${\text{V}} = \left( {{\text{L}} \cdot {\text{W}}^{2} } \right)/2$, which took an average of 10 ± 4 days.

### Control and experimental groups

Balb/c mice were randomly divided into five groups once tumors reached 75 mm^3^. The first group (n = 16) received saline injections as a control. The second group (n = 15) received an isotype control. The third group received anti-CCL2 only (n = 14). The fourth group (n = 14) received 5-FU only. The fifth group (n = 13) received a combination of 5-FU and anti-CCL2. Mice were euthanized via cervical dislocation if they reached humane end-point euthanasia criteria as specified by the University of Arkansas IACUC (maximum tumor diameter ≥ 20 mm, body condition score ≤ 2, weight loss ≥ 20% from baseline, macroscopic evidence of ulceration or chronic pain or distress detailed by the University of Rhode Island IACUC.

### Chemotherapy

5-Fluorouracil (5-FU), purchased from Sigma-Aldrich (St. Louis, MO, USA), is an antitumor chemotherapy agent that induces p53-dependent apoptosis and decreases proliferation [31]. 5-FU powder (Sigma Aldrich, #F6627-10G) was diluted in DMSO at 40 mg/mL and stored at − 20 °C for a maximum of two months before injection. A second dilution of 5-FU/DMSO was created in sterile saline (VWR, Radnor, PA, #89167-774) to bring the 5-FU concentration to 20 mg/mL. On the day of injection, aliquots of 20 mg/mL 5-FU + DMSO in sterile saline were further diluted to 3 mg/mL 5-FU/saline in sterile microcentrifuge tubes (VWR, #20,170–038), and brought to 37.3 °C. Using a 28G insulin syringe (VWR, #BD329410), mice in the 5-FU and combination groups received daily intraperitoneal (IP) administration of 5-FU at a concentration of 15 mg/kg/dose [32] until day 10 (165 mg/kg/week). This resulted in an average injection of 300 µg 5-FU in 100 µL vehicle, based on average mouse weight of approximately 20 g at the time of injection.

### Immunotherapy

Anti-CCL2 (clone number: 2H5), purchased from Bio X Cell (West Lebanon, NH, USA), is a monoclonal antibody that neutralizes murine CCL2 [31, 33]. Anti-CCL2 (Bio X Cell, 2H5, #BE0185) was shipped at 7.4 mg/mL in PBS and stored at 4 °C before injection. On the day of injection, aliquots of 7.4 mg/mL anti-CCL2/PBS solution were diluted with sterile saline (VWR, #89167-774) to 1 mg/mL anti-CCL2/saline in sterile microcentrifuge tubes (VWR, #20170-038), and brought to 37.3 °C. Using a 28G insulin syringe (VWR, #BD329410), anti-CCL2 and combination groups received IP administration of anti-CCL2 at a concentration of 4.0 mg/kg/dose [28, 31] given every other day through day 10 (24 mg/kg/week). This resulted in an average injection of 80 µg anti-CCL2 (2H5) in 100 µL vehicle, based on average mouse weight of 20 g at the time of injection.

### Isotype control

An Armenian hamster IgG isotype control, purchased from Bio X Cell (West Lebanon, NH, USA), is a polyclonal antibody that is ideal for its use as a non-reactive control. The isotype control (IgG) (Bio X Cell, #BE0091) was shipped at 7.4 mg/mL in PBS and stored at 4 °C before injection. On the day of injection, aliquots of 7.4 mg/mL IgG/PBS solution were diluted with sterile saline (VWR, #89167-774) to 1 mg/mL IgG/saline in sterile microcentrifuge tubes (VWR, #20170-038), and brought to 37.3 °C. Using a 28G insulin syringe (VWR, #BD329410), the isotype group received IP administration of IgG at a concentration of 4.0 mg/kg/dose [28, 31] given every other day through day 10 (24 mg/kg/week). This resulted in an average injection of 80 µg IgG in 100 µL vehicle, based on average mouse weight of 20 g at the time of injection.

### Diffuse reflectance spectroscopy

#### DRS instrumentation

**Fig. 1:**
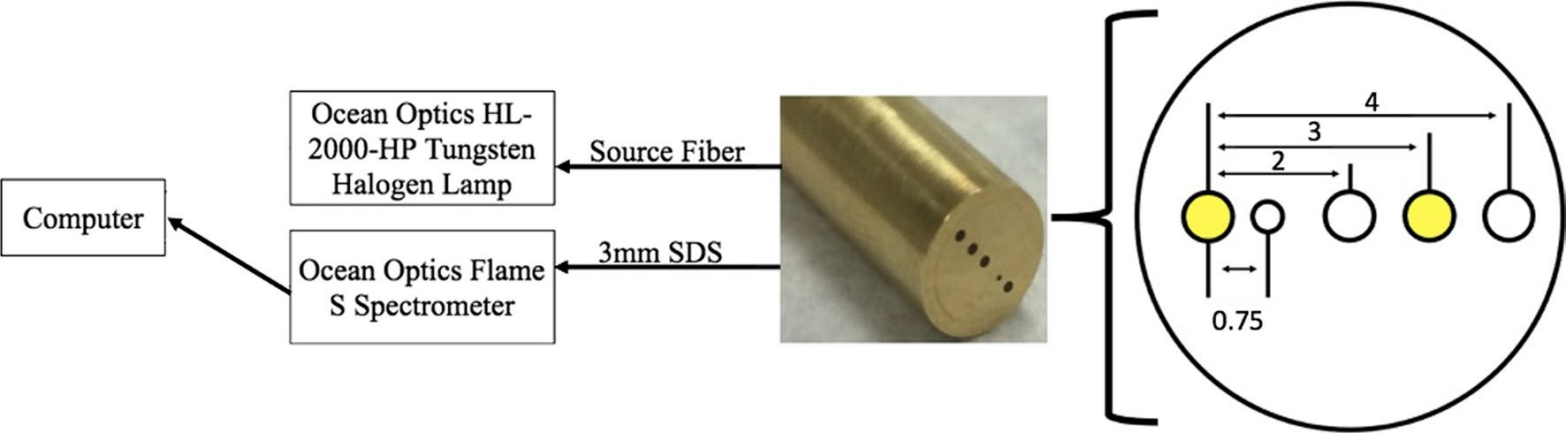
Schematic of DRS system. The DRS probe was connected to an Ocean Optics Flame S spectrometer through the 3 mm SDS and to an Ocean Optics HL-2000-HP Tungsten Halogen Lamp through the source fiber. The halogen lamp and spectrometer were connected to a computer display with a custom LABView software program to collect spectra and calibration measurements

The DRS probe (FiberTech Optica, Kitchener, ON, Canada) was 1.0 m in total length, with the split position located 0.67 m from the common (distal) end. Five individual optical fibers (one source and four detectors) were integrated within the distal brass ferrule (6.35 mm diameter × 50 mm long). All fibers were arranged linearly in a slit line, resulting in source-detector separations (SDS) of 0.75, 2.00, 3.00, and 4.00 mm. Each multimodal optical fiber consisted of a high-OH silica core, a silica cladding, and a polyimide jacket optimized for a wavelength range of 190–1200 nm, which exceeded the desired wavelength range of 450–750 nm used in this study. The source fiber, as well as the 2.00, 3.00, and 4.00 SDS fibers (FiberTech Optica, SUV400/440PI) consisted of a 400/440 μm ± 2% silica core/cladding with a 470 μm ± 5% polyimide jacket. The 0.75 mm SDS fiber (FiberTech Optica, SUV200/220PI) consisted of a 200/220 μm ± 2% silica core/cladding with a 245 μm ± 5% polyimide jacket. These optical fibers were included to sample into the subcutaneous murine CT26 tumor at multiple sampling depths to quantify THC, StO_2_, and HbO_2_, and dHb. A 20 W tungsten-halogen lamp (Ocean Optics, HL-2000-HP) provided broadband light (360–2400 nm) to the 400 μm core source fiber. A spectrometer (Ocean Optics, FLAME-S) with a Sony ILX511B linear silicon CCD array (2,048-pixel elements) collected diffusely reflected light from the 3 mm SDS (Fig. 1).

#### Diffuse reflectance spectroscopy measurements

**Fig. 2:**
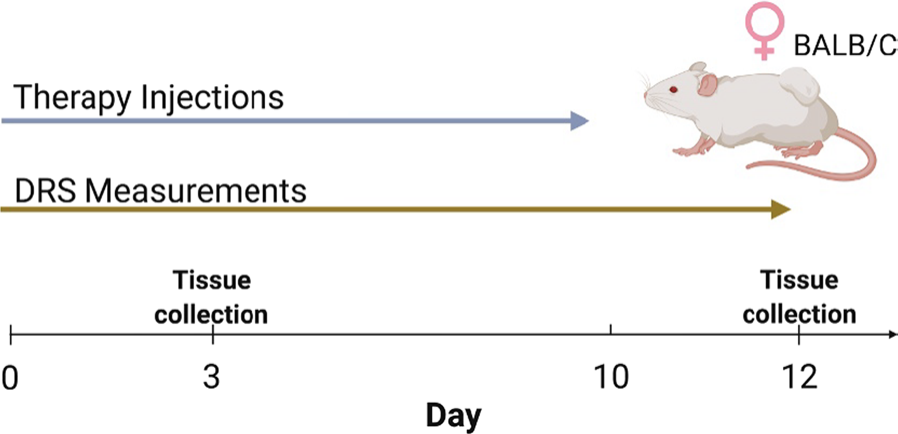
Timeline for study. Daily DRS measurements were taken after tumor volumes reached 75 mm^3^ up until endpoint analysis (Day 3 or 12). Therapy injections were given on days 0 to 10. 5-FU treated mice were given daily injections while the other treatment groups (saline and isotype controls and anti-CCL2) were given injections every other day. The combination group followed the treatment schedule of the anti-CCL2 and 5-FU groups. Figure was created with BioRender.com

All mice (n = 95) underwent DRS measurements starting at day 0. The hair on the tumor site was removed via depilatory lotion and then cleaned 24 h prior to the first DRS measurement. In this study, only the 3.00 mm SDS was used. The 3.00 mm SDS provided an optimal balance of signal-to-noise (> 15 dB), appropriate in vivo integration time (70 ms), and wavelength-dependent sampling depth (~ 1.3 to 2.1 mm). DRS measurements were taken daily until the mice reach endpoint IHC analysis (Fig. 2).

The DRS probe was placed on the tumor such that the linear arrangement of optical fibers was collinear with the long axis of the tumor (cranial to caudal direction) of non-anesthetized mice while spectra were collected. For each tumor, ≥ 20 DRS measurements were acquired at an integration time of 70 ms. Day-to-day fluctuations in light source intensity were controlled by calibration with a Spectralon® 20% diffuse reflectance standard. Daily spectrometer dark noise was subtracted from each spectra.

Each DRS measurement resulted in a value for THC, StO_2_, HbO_2_, dHb (630 nm) [34], and a chi-square (χ^2^) value. THC, StO_2_, HbO_2_, dHb were quantified by inputting raw DRS spectra into custom lookup-table (LUT)-based MATLAB software with a priori values for oxygenated and deoxygenated hemoglobin extinction coefficients [35–37]. The software performed an iterative model fit (1 × 10^4^ iterations) to the raw DRS data to quantify THC, StO_2_, HbO_2_, dHb. The χ^2^ value indicated goodness-of-fit between the model fit and raw DRS data; high χ^2^ values usually implied specular reflection due to user movement. Therefore, if χ^2^ of the spectra exceeded 10, data were discarded. THC, StO_2_, HbO_2_, dHb values were averaged to yield a daily result.

### Immunohistochemistry and quantification

Tumors were placed in a 50 mL conical centrifuge tube of 10% neutral buffered formalin for 24 h at 4 °C. After 24 h, formalin was replaced with 50 mL phosphate-buffered saline (PBS), then placed in 50 mL 70% ethanol before shipment (IHC World, Ellicott City, MD, USA) for IHC staining. Tumors were paraffin-embedded, sectioned in 5 µm sections, and stained for anti-CD68 (Abcam®, ab125212), anti-CD163 (Abcam®, ab182422), anti-carbonic anhydrase-9 (CA-IX) (Abcam®, ab184006), anti-CD31 (Abcam®, ab28364), and H&E.

#### Total-tumor-associated macrophage recruitment

anti-CD68 stained slides were imaged with a wide-field upright microscope (Nikon, Eclipse Ci), with a 40X/1.30NA oil objective lens (Nikon, CFI Plan Fluor 60X), digital camera (Nikon, DS-Fi2), and PC-based camera control unit (Nikon, DS-U3). For each mouse, three 5 μm tissue sections were imaged and nine high-powered fields-of-view (FOV: 50 µm^2^) were taken for each of the three sections for a total of nine TAM FOVs per tumor. In ImageJ, images were converted to 8-bit and thresholded using the average pixel intensity of a positively stained TAM using Otsu’s method. All counts were made blinded to DRS data. All positively stained cells were counted over the nine FOVs to calculate the average TAM count per FOV.

#### M2-tumor-associated macrophage recruitment

anti-CD163 stained slides were imaged with a wide-field upright microscope (Nikon, Eclipse Ci), with a 40X/1.30NA oil objective lens (Nikon, CFI Plan Fluor 60X), digital camera (Nikon, DS-Fi2), and PC-based camera control unit (Nikon, DS-U3). For each tumor, three 5 μm tissue sections were imaged and three high-powered fields-of-view (FOV: 50 µm^2^) were taken for each of the three sections for a total of nine TAM FOVs per tumor. In ImageJ, images were converted to 8-bit and the color channels were separated (brown vs blue). Images containing the positive brown staining were thresholded using the average pixel intensity of a positively stained TAM using Otsu’s method. All counts were made blinded to DRS data. All positively stained cells were counted over the nine FOVs to calculate the average TAM count per FOV.

#### Hypoxia

Slides stained for anti-CA-IX were imaged with a wide-field upright microscope (Nikon, Eclipse Ci), 10X/0.3NA objective lens (Nikon, CFI Plan Fluor 10X), digital camera (Nikon, DS-Fi2), and PC-based camera control unit (Nikon, DS-U3) [38]. For each tumor, three 5 μm thick tissue sections were imaged. Three high-powered fields-of-view (FOV: 200 µm^2^) were taken for each of the three sections for a total of nine FOVs per tumor. In ImageJ, images were thresholded using “Color Threshold” based on the darkest pixel color (brown). The hypoxic area for each image was calculated using this formula:

$${\text{Hypoxic}}\,{\text{area}} = \frac{{{\text{Positive}}\,{\text{CA}} - {\text{IX}}\,{\text{stained}}\,{\text{area}} \left( {{\text{pixels}}} \right)}}{{{\text{Total}}\,{\text{area}}\,\left( {{\text{pixels}}} \right)}} \times 100\%$$

where the numerator represents the total number of pixels that were captured using the “Color Threshold” and the denominator represents the total number of pixels within the image. All positively stained areas were counted over the nine FOVs to calculate the average hypoxic area.

#### Vasculature

Slides stained for anti-CD31 were imaged with a wide-field upright microscope (Nikon, Eclipse Ci), 10X/0.3NA objective lens (Nikon, CFI Plan Fluor 10X), digital camera (Nikon, DS-Fi2), and PC-based camera control unit (Nikon, DS-U3). For each tumor, three 5 μm thick tissue sections were imaged. Three high-powered fields-of-view (FOV: 200 µm^2^) were taken for each of the three sections for a total of nine FOVs per tumor. In ImageJ, images were thresholded using “Color Threshold” based on the darkest pixel color (brown). The vascular density for each image was calculated using this formula [39]:

$${\text{Vascular}}\,{\text{Density}} = \frac{{{\text{Positive}}\,CD31\,{\text{stained}}\,{\text{area}} \left( {{\text{pixels}}} \right)}}{{{\text{Total}}\,{\text{area}} \left( {{\text{pixels}}} \right)}} \times 100\%$$

where the numerator represents the total number of pixels that were captured using the “Color Threshold” and the denominator represents the total number of pixels within the image. All positively stained areas were counted over the nine FOVs to calculate the average vascular density.

### Statistical analysis

The significance of datasets was set to *p* < 0.05. Statistical analysis was performed using Prism (GraphPad, version 8.4.3 (471)). A mixed-effects model with a Dunnett’s multiple comparisons test was used to assess statistical differences for the tumor volume and DRS data. Simple linear regression was used to determine the goodness of fit for the correlation plots.

## Results

### Combination therapy shows improvement in tumor volume survival and slows tumor growth

**Fig. 3:**
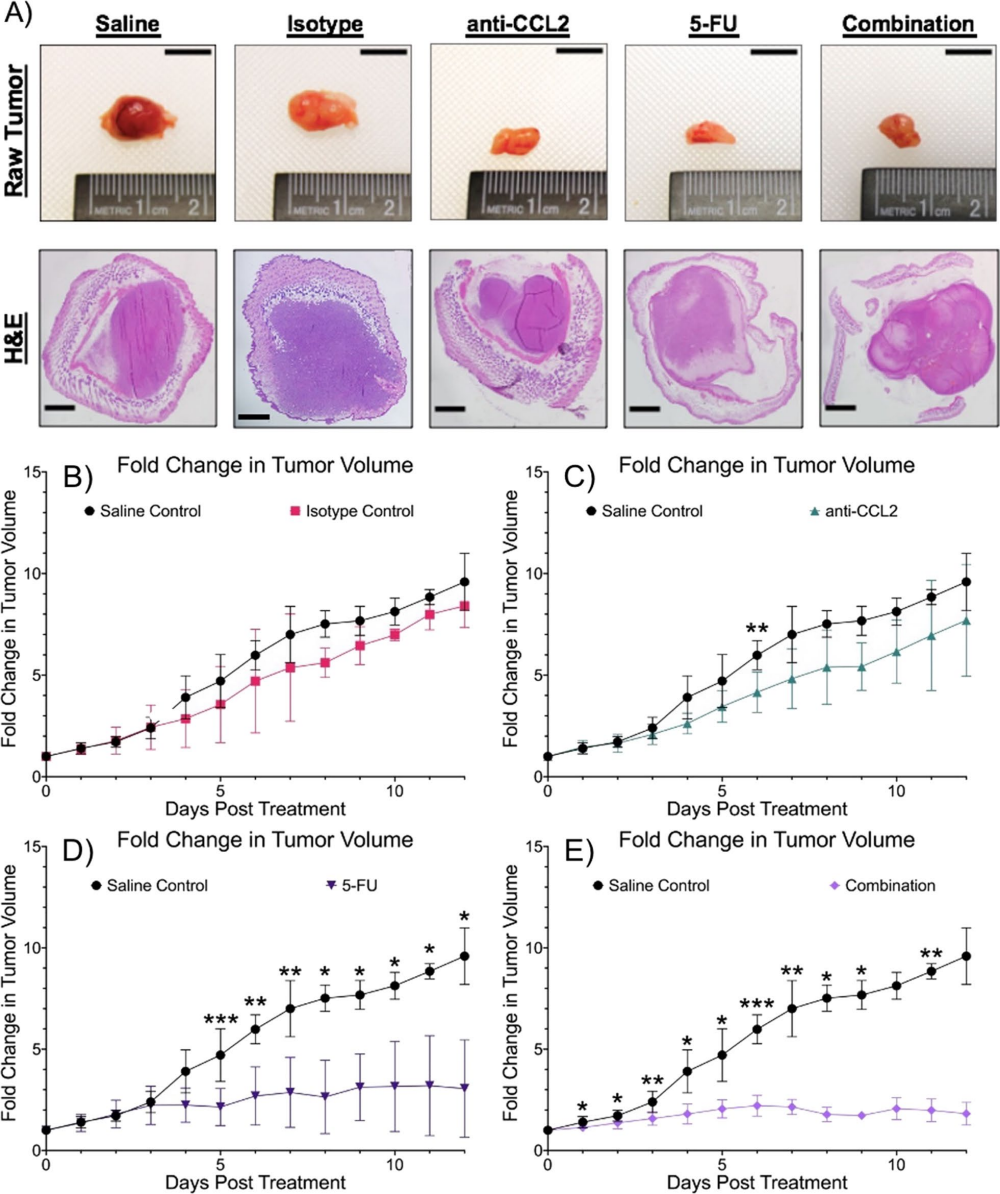
Combination therapy shows slowed tumor growth. **A** Raw tumors after resection (scale bars are 10 mm) and representative H&E images (4.0X objective, 0.25NA scale bars are 100 μm). **B**–**E** Longitudinal comparisons of fold changes in tumor volume of a twelve-day duration. A mixed effects model was used to calculate statistical differences (Saline vs Combination: *p ≤ 0.05, **p ≤ 0.01, ***p ≤ 0.001, ****p ≤ 0.0001. Plots created in Prism (GraphPad)

To study the effects of combinatorial therapy, tumor volumes were compared between each experimental group. Tumor volumes were measured daily after the initial threshold of 75 mm^3^ was reached. Figure 3A shows representative images of resected tumors and H&E-stained tumors for each group. These results suggest that the addition of anti-CCL2 to 5-FU decreases tumor volume over time. In Fig. 3B–E., the average fold-change in tumor volume between groups was explored during our twelve-day study. The isotype and saline controls show a similar fold change in tumor volume across the duration of treatment. In the anti-CCL2 group, there was a smaller fold change in tumor volume with one significant difference observed on Day 6 (*p* = 0.0089). The 5-FU showed a smaller fold change in tumor volume than the control groups and the anti-CCL2 group. Significant differences were observed on Days 5 through 12 with greatest difference in fold change in tumor volume observed on Day 5 (*p* = 0.0003) when compared to the saline control. The combination group showed the greatest difference in the average fold change in tumor volume during the course of treatment, with exception to Days 10 and 12. The greatest difference in the fold change in tumor volume between the saline group and the combination group was seen on Day 6 (*p* < 0.0001). This trend between these two groups continues through the duration of the study. Overall, throughout the duration of the study, the combination therapy showed aa approximately one to two-fold change (change from the original measurement to the final measurement) decrease in tumor growth.

### Anti-CCL2 significantly reduces TAM recruitment into the tumor microenvironment

**Fig. 4:**
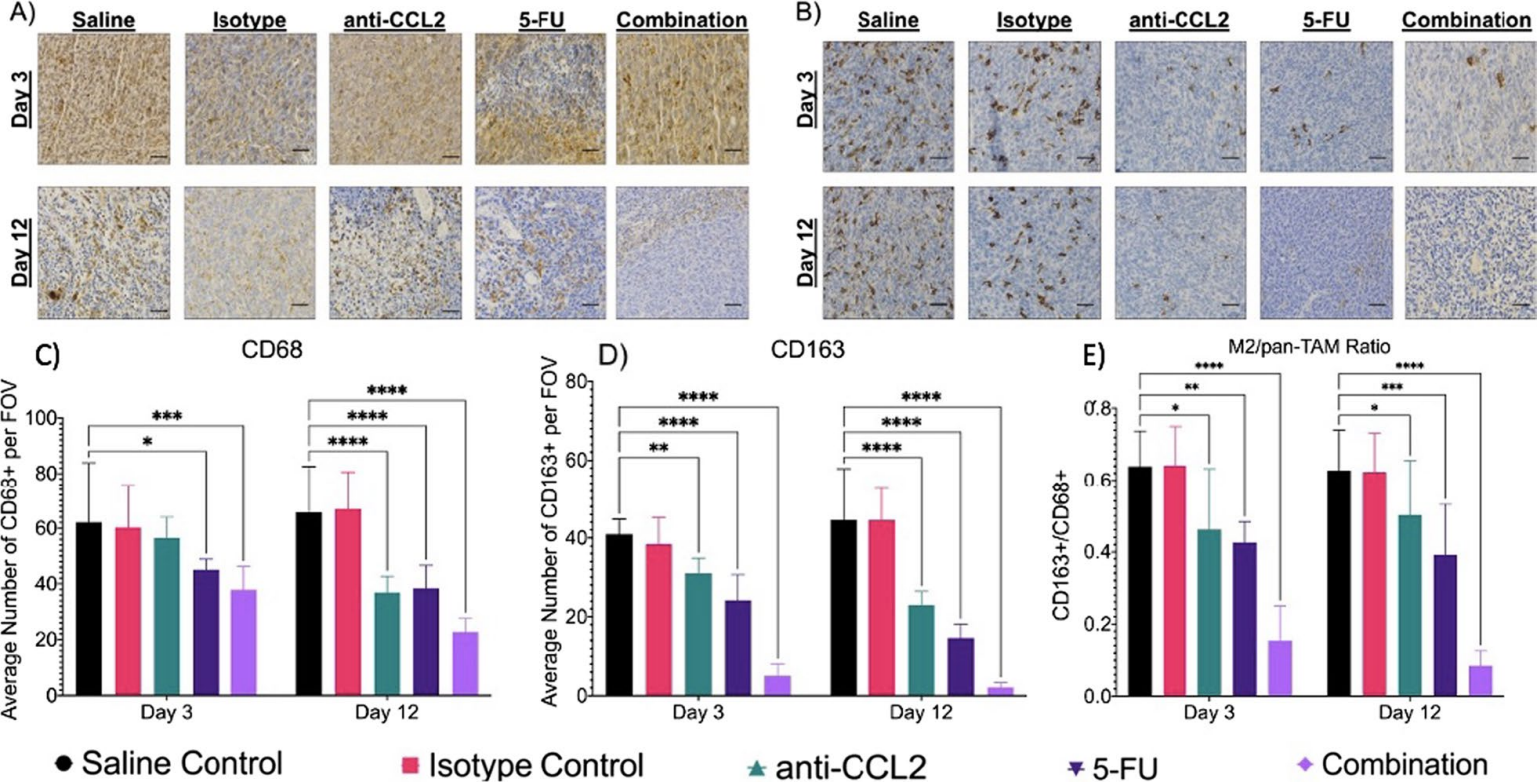
Combination therapy shows decrease in total and M2 macrophage recruitment. **A** Total TAM recruitment through IHC quantification using CD68 (40X, scale bars are 20 μm). **B** M2 TAM recruitment through IHC quantification using CD163. The average number of positively stained **C** CD68 (n = 27 FOVs per group) and **D** CD163 (n = 27 FOVs per group). TAMs were counted per FOV. Then the **E** ratio of M2 TAMs to total TAMs was calculated. A mixed effects model was used to calculate statistical differences (*p ≤ 0.05, **p ≤ 0.01, ***p ≤ 0.001, ****p ≤ 0.0001). Plots created in Prism (GraphPad)

To test the effect of anti-CCL2 on TAM recruitment, subcutaneous tumors were labeled with a pan-TAM marker, CD68. When comparing the macrophage recruitment between groups, the combination group showed the greatest decrease in TAMs between days three and twelve (Fig. 4A, C). For the saline and isotype controls, both groups showed similar results for total macrophage recruitment for both days 3 and 12. While imaging the isotype group on day 12, the intensity of the brown pixels (CD68 marker) was fainter than that of the saline control. The intensity of these pixels did not affect the overall result for total macrophage recruitment. After three days of treatment, the control groups showed similar results while the anti-CCL2 group showed a modest decrease (~ 10%) in total TAM recruitment. When compared to the saline control, the 5-FU group showed a decrease of ~ 20% in total TAM recruitment with a significant difference when compared to the saline control (*p* = 0.0159) while the combination group showed a major decrease in total TAM recruitment (~ 25%) with a significant difference when compared to the saline control (*p* = 0.0003). On day twelve, again, the controls showed similar results while the anti-CCL2, 5-FU, and combination groups showed a significant difference (*p* < 0.0001 for all groups). The anti-CCL2 showed an ~ 20% decrease in total TAM recruitment from day three to twelve. The 5-FU group showed an ~ 15% decrease in total TAM recruitment from day three to twelve. Finally, the combination group showed an ~ 20% decrease in total TAM recruitment from day three to twelve. Overall, results show that the addition of anti-CCL2 to 5-FU shows a significant decrease in total TAM recruitment into the microenvironment.

Since TAM infiltration increases pro-tumor functions such as angiogenesis and tumor burden, we wanted to look at the recruitment of M2-phenotype TAMs into the tumor microenvironment using CD163 (a common M2 marker). Similar to the results shown in Fig. 4A, the combination group shows the greatest decrease in the recruitment of M2 TAMs. Significant differences in M2 TAM infiltration were observed in the treatment groups on days three and twelve when compared to the saline control (Fig. 4B, D). On day 3, anti-CCL2 treated tumors showed a modest decrease (~ 20%) in M2 TAMs (*p* = 0.004), while the 5-FU and combination groups showed major differences (~ 15 to 30% decrease) in M2 TAM recruitment (*p* < 0.0001 for both groups). On day twelve, major differences were observed in the anti-CCL2 (a decrease of ~ 20%) (*p* < 0.0001) and 5-FU (a decrease of ~ 20%) (*p* < 0.0001) groups with the combination group showing the greatest difference (a decrease of ~ 35%) (*p* < 0.0001). The anti-CCL2 showed an ~ 10% decrease in M2 TAM recruitment from day three to twelve. The 5-FU group showed an ~ 15% decrease in M2 TAM recruitment from day three to twelve. Finally, the combination group showed an ~ 10% decrease in M2 TAM recruitment from day three to twelve. Overall, results show that the addition of anti-CCL2 to 5-FU shows a significant decrease in M2 TAM recruitment into the microenvironment.

Lastly, we examined the ratio of the number of M2 to total TAMs being recruited into the tumor. The control groups showed the highest M2/pan-TAM ratio at ~ 0.6 (Fig. 4E). This ratio remained approximately the same for the anti-CCL2 for both days (~ 0.5) while the 5-FU group showing a slight decrease (~ 0.4 to 0.35) and the combination group showing the lowest ratio (~ 0.18 to ~ 0.15). After three days, the anti-CCL2 showed a slight difference compared to the saline control (*p* = 0.01) with the 5-FU showing a significant difference (*p* = 0.0015) and the combination group showing the greatest difference (*p* < 0.0001). Similar results are observed on day twelve (*p* = 0.0462, *p* = 0.0004, *p* < 0.0001, respectively). Taken together with the results shown in Fig. 4C, D, results indicate that the addition of anti-CCL2 to 5-FU decreases TAM infiltration into the tumor microenvironment including the TAM phenotype that induces pro-tumor function.

### DRS-derived metrics quantify tumor response during treatment

To investigate the effects of combination or immunotherapy during the duration of treatment, we compared DRS-derived metrics oxygen saturation (StO_2_), total hemoglobin content (THC), oxyhemoglobin (HbO_2_), and deoxyhemoglobin (dHb) between groups.

**Fig. 5:**
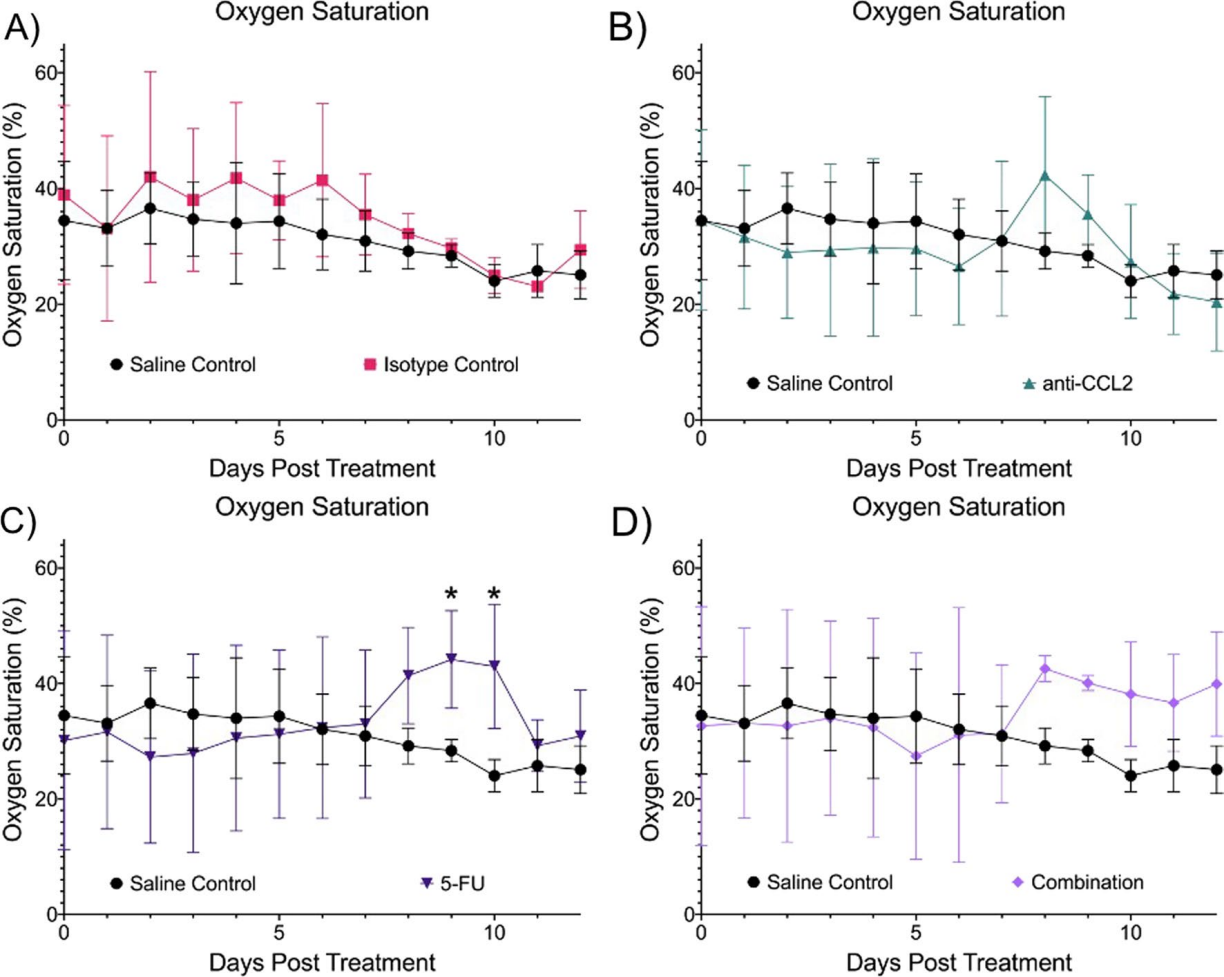
Longitudinal comparisons in oxygen saturation. Each panel shows the comparison of oxygen saturation in comparison to the saline and isotype control. **A** Isotype control, **B** anti-CCL2, **C** 5-FU and **D** Combination. A mixed effects model was used to calculate statistical differences (*p ≤ 0.05). Plots created in Prism (GraphPad)

Figure 5 shows the longitudinal comparisons in StO_2_ between the treatment groups and the saline control. The isotype control (Fig. 5A) showed a comparable StO_2_ level (~ 5–10%) with that of the saline control throughout the course of treatment. The anti-CCL2 group (Fig. 5B) showed no significant differences in StO_2_ levels compared to the saline control. A small increase in StO_2_ was observed on Days 7 through 9. The 5-FU group (Fig. 5C) also showed two significant differences in StO_2_ levels when compared to the saline control even though the StO_2_ levels were higher than the saline and isotype controls on Days 9 and 10 (*p* = 0.0305 and *p* = 0.0391, respectively). The combination group (Fig. 5D) showed higher StO_2_ starting seven days after initial treatment. There were no significant differences in StO_2_ when compared to the saline control.

**Fig. 6:**
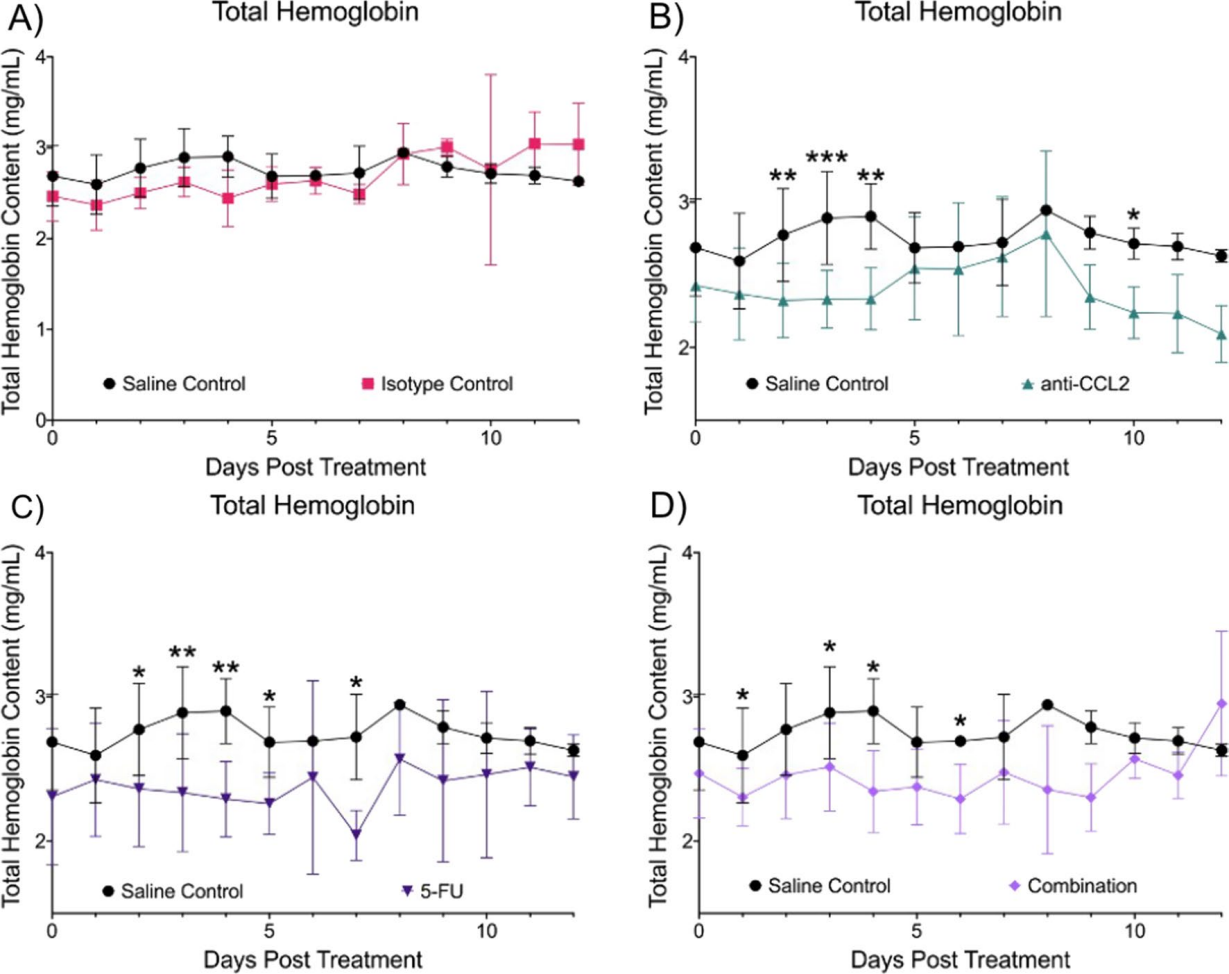
Longitudinal comparisons in total hemoglobin content. Each panel shows the comparison of oxygen saturation in comparison to the saline and isotype control. **A** Isotype control, **B** anti-CCL2, **C** 5-FU and **D** Combination. A mixed effects model was used to calculate statistical differences (*p ≤ 0.05, **p ≤ 0.01, ***p ≤ 0.001). Plots created in Prism (GraphPad)

Next, we compared the THC between groups (Fig. 6). The isotype group (Fig. 6A) had comparable THC levels with the saline control group throughout the course of treatment. The anti-CCL2 treated group (Fig. 6B) had consistently lower THC levels throughout the course of treatment. There were several significant differences observed in THC between the anti-CCL2 group and the saline control (Day 2, *p* = 0.0041; Day 3, *p* = 0.0004; Day 4, *p* = 0.00398; and Day 10, *p* = 0.0465). Like the anti-CCL2 group, the 5-FU group (Fig. 6C) also showed consistently lower levels of THC across the course of treatment. There were five days where the THC levels were significantly different (Day 2, *p* = 0.0333; Day 3, *p* = 0.0041; Day 4, *p* = 0.0035; Day 5, *p* = 0.0203, and Day 7, *p* = 0.0109). The combination group (Fig. 6D) showed significant differences of THC levels on Day 1 (*p* = 0.0251), Day 3 (*p* = 0.0394), and Day 4 (*p* = 0.0110).

HbO_2_ was calculated using the following formula: ${\text{HbO}}_{2} = {\text{StO}}_{2} {\text{*THC}}$, where StO_2_ is the oxygen saturation and THC is the total hemoglobin in the tumor volume. The saline and isotype controls showed comparable levels of HbO_2_ (Additional file 1: Fig. S1A). The anti-CCL2 group showed slightly lower levels of HbO_2_ throughout the treatment course except for Day 8 where there was a spike in HbO_2_. Significant differences were observed on Days 2 and 3 (*p* = 0.0239 and *p* = 0.0467, respectively). (Additional file 1: Fig. S1B). The 5-FU group showed varying levels of HbO_2_ with increases in HbO_2_ on Day 8 through 12 (Additional file 1: Fig. S1C). The combination group also showed varying levels of HbO_2_ with an increase in HbO_2_ after Day 8 (Additional file 1: Fig. S1D). No significant differences were observed.

Lastly, we investigated the differences in dHb (Additional file 1: Fig. S2). dHb was calculated using the following formula: ${\text{dHb}} = {\text{ THC}} - {\text{HbO}}_{2}$, where THC is the total hemoglobin and HbO_2_ is the oxyhemoglobin in the tumor volume. The saline and isotype controls showed similar levels of dHb across the course of treatment (Additional file 1: Fig. S2A). The anti-CCL2 treated group showed slight lower dHb levels when compared to the saline control. One difference was observed on Day 8 (*p* = 0.0210) (Additional file 1: Fig. S2B). The 5-FU and combination groups also showed consistently lower dHb levels across the treatment course. (Additional file 1: Fig. S2C and 2D). In the 5-FU group, two significant differences were observed on Days 7 through 9 (*p* = 0.0049, *p* = 0.0133, and *p* = 0.0334, respectively). One significant difference was observed on day 8 for the combination group (*p* = 0.0303).

### Hypoxia expression in subcutaneous murine CRC tumors

**Fig. 7:**
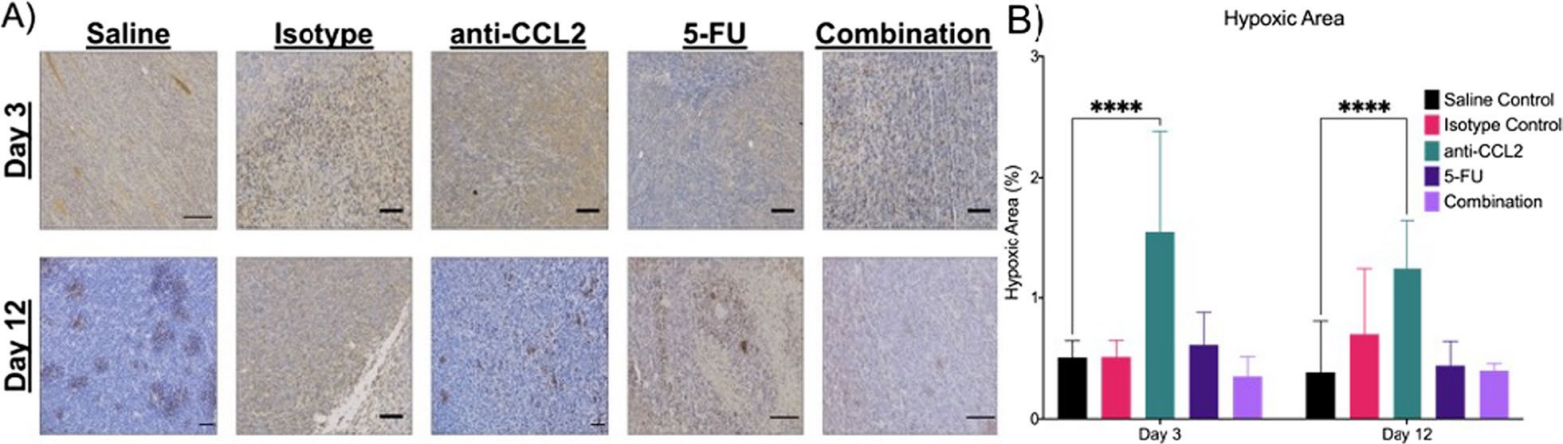
The addition of anti-CCL2 to 5-FU shows a change in hypoxia. **A** Hypoxia quantification through IHC quantification using CA-IX (10X objective, 0.3NA, scale bars are 50 μm). **B** The average hypoxic area was calculated per FOV (n = 27 FOVs per group). A mixed effects model was used to calculate statistical differences. Plot created in Prism (GraphPad)

Immunohistochemical analysis using the marker CA-IX was used to explore the differences in hypoxia between experimental groups. In Fig. 7A, representative images are shown to visually compare the amount of hypoxia in a limited tumor volume. From 9 FOVs per tumor (n = 3 per group), the hypoxic area was calculated. After three days, one major significant difference was observed. The anti-CCL2 group showed the greatest hypoxic area (~ 1.6%) compared to the other treatment groups, especially the saline control (*p* < 0.0001). The 5-FU group showed a slightly higher hypoxic area (~ 0.6%) when compared to the controls while the combination group showed a slightly lower hypoxic area (~ 0.4%). After twelve days, the anti-CCL2 still shows the highest hypoxic area of the treatment groups (~ 1.25%), but the isotype group does show an increase of ~ 0.5% in the hypoxic area compared to day three. Significant differences were observed in the anti-CCL2 (*p* < 0.0001) compared to the saline control on Days 3 and 12. The 5-FU group showed a slightly higher hypoxic area (~ 0.6%) when compared to the saline control on Day 3. Based on these results, the hypoxic area of localized tumors in the combination group was lower than that of the anti-CCL2 and 5-FU groups after three days of treatment and remains comparable to that of the 5-FU group after twelve days of treatment.

### Changes in vascular density in subcutaneous murine CRC tumors

**Fig. 8:**
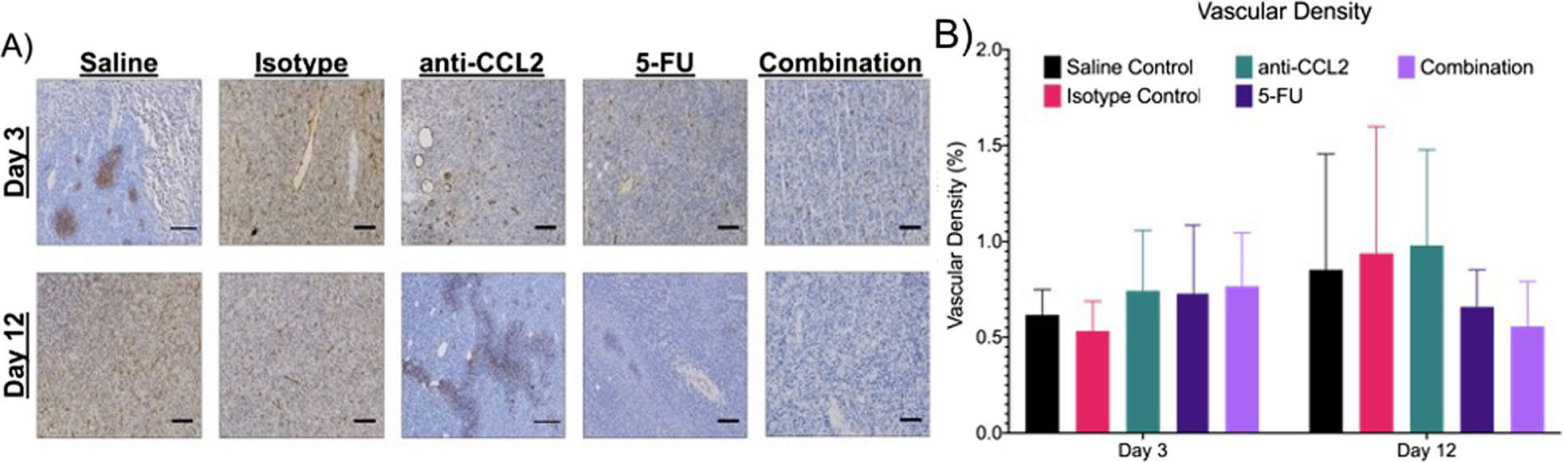
The combination therapy regimen shows a decrease in new blood vessel formation. **A** Blood vessel quantification through IHC quantification using CD31 (10X objective, 0.3NA, scale bars are 50 μm). **B** The average vascular density was calculated per FOV (n = 27 FOVs per group). A mixed effects model was used to calculate statistical differences. Plot created in Prism (GraphPad)

Immunohistochemical analysis using the marker CD31 was used to explore the differences in vascular density between groups. In Fig. 8A, representative images are shown to visually compare the amount of hypoxia in a limited tumor volume. From 9 FOVs per tumor (n = 3 per group), the hypoxic area was calculated. After three days of treatment, the anti-CCL2, 5-FU and combination groups also showed a higher density of blood vessels, but only a slight increase (~ 0.25%) compared to the saline control. After twelve days, the saline and isotype control groups along with the anti-CCL2 group showed an increase in microvasculature (~ 0.3 to 0.4%) while the 5-FU and combination treatment showed a decrease (~ 0.2%). The combination group showed the greatest decrease in the density of blood vessels on day twelve.

### Validation of DRS metrics through IHC correlations

**Fig. 9:**
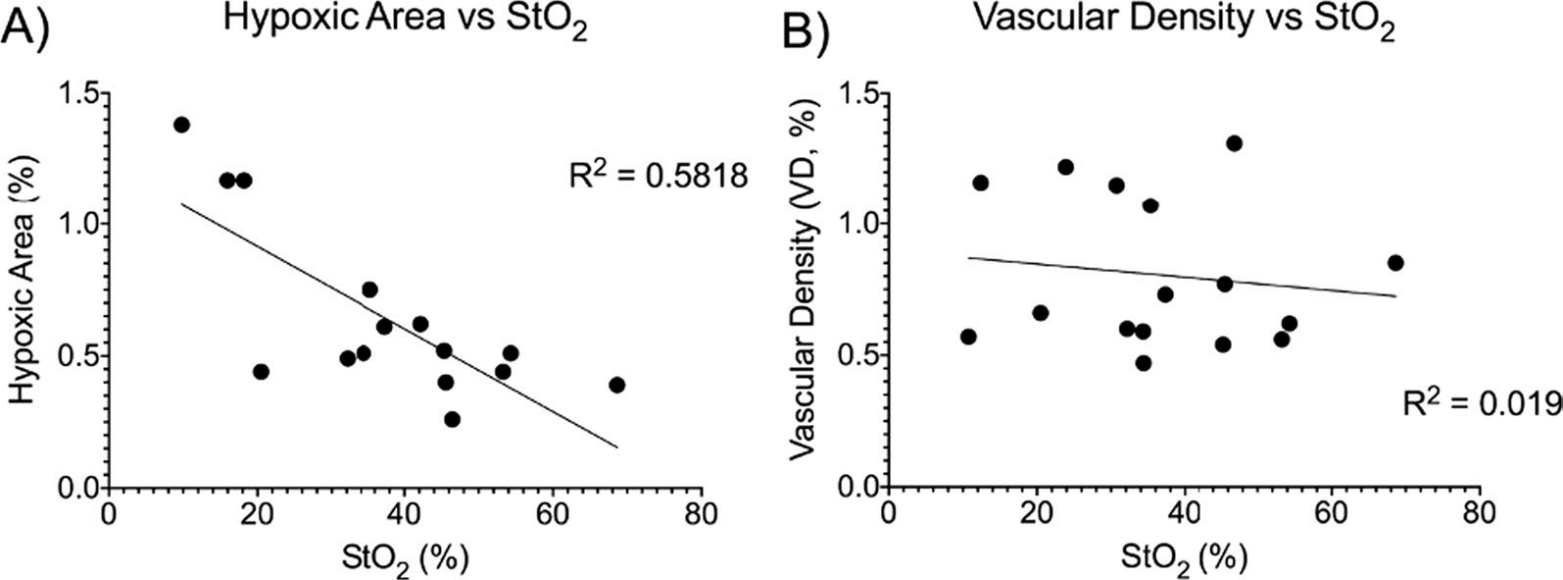
Hypoxic area shows a moderate negative correlation to DRS-derived tumor oxygenation. Correlations comparing DRS-derived tumor oxygenation to **A** hypoxic area and **B** vascular density. A simple linear regression was performed to produce a goodness of fit (R^2^). Plots created in Prism (GraphPad)

Since DRS is a new minimally invasive tool to monitor treatment response, we wanted to see if there was a correlation between the DRS-derived metrics and a trusted immunohistochemistry marker. In Fig. 9, correlation plots were created to investigate the relationship between DRS-derived oxygenation and hypoxic area (Fig. 9A) and vascular density (Fig. 9B). There is a moderate negative correlation between hypoxic area and oxygenation, as shown in Fig. 9A (R^2^ = 0.5818). This indicates that the higher the hypoxic area, the lower the oxygenation, which is consistent with the literature. In Fig. 9B, there is a weak correlation between vascular density and oxygenation. The correlation between hypoxic area and oxygenation shows that DRS can be used to monitor treatment response.

## Discussion

Before advancements in cancer detection and treatment, conventional treatment options for locally advanced CRC include adjuvant and neoadjuvant chemotherapy and radiotherapy. Although these treatment regimens have been shown to decrease tumor burden, high recurrence rates are still a major limitation [25]. In recent years, immunotherapy strategies have shown promise in the reduction of the high recurrence rate while minimizing undesirable side effects. Various clinical trials that target vascular endothelial growth factors (VEGFs) or epidermal growth factors (EGFs) in several cancer types such as breast cancer have shown clinical success [25]. In CRC, the overall survival benefit is limited [25]. Here, we used chemokine neutralization via the CCL2 pathway to target circulating monocytes that differentiate into M2 pro-tumor TAMs.

Chemokines are involved in inducing cellular chemotaxis, making them an attractive target for CRC therapy [25]. One pathway that is involved is the CCL2 pathway. CRC recruits circulating monocytes through the release of CCL2. In the tumor microenvironment, these monocytes differentiate into tumor-associated macrophages (TAMs), which have a pervasive effect on immune cells in the microenvironment. TAMs have both anti-tumor and pro-tumor functions depending on their polarization. M2-polarized TAMs (pro-tumor) functions include immunosuppression, angiogenesis, tumor growth, increased vascular permeability, and metastatic risk [38]. Targeting of CCL2 to reduce M2-polarized TAMs is an attractive strategy in pre-clinical settings. In this study, we investigated the downstream effects of CCL2. Even though there were no significant differences in the change in tumor volume after three days, there were significant changes twelve days after the start of treatment. The blockade of CCL2 alone showed a slowing in the overall fold change in tumor volume (~ threefold) compared to a saline control twelve days after the initiation of treatment. However, when anti-CCL2 was added to the gold standard 5-FU, the overall fold change in tumor growth decreased significantly (~ sevenfold) compared to a saline control twelve days post-treatment. The blockade of CCL2 also showed a significant decrease in the number of TAMs recruited into the tumor microenvironment. This shows that CCL2-neutralizing immunotherapy can play an important role in early-stage CRC treatment.

Tumor oxygenation and angiogenesis have been widely studied and have been shown to have an effect on patient prognosis. Angiogenesis favors tumor growth and metastasis, while hypoxia promotes resistance to chemotherapy [38]. Tumor cells are able to adapt to hypoxic conditions through the stabilization of hypoxia-inducible transcription factor (HIF-1α), which leads to the upregulation of several genes involved in cellular proliferation and angiogenesis. One of these genes includes carbonic anhydrase (CA-IX). As mentioned in Korkeila et al. [38], patients with hypoxic tumors had poorer disease outcomes. Preoperative treatment that has slowed tumor growth can also show an increase in tumor oxygenation. Hence, preoperative therapy may reduce tumor hypoxia and therefore reduce CA-IX expression [38]. In this study, we used CA-IX to study hypoxia expression and CD31 to study vessel density after treatment with immunomodulation therapy. Alone and in combination with 5-FU, the blockade of CCL2 did show a decrease in the overall expression of CA-IX. After twelve days, the combination group showed the greatest decrease in vessel density when compared to a control. When comparing the hypoxic areas and vessel densities of the anti-CCL2 group, we found that hypoxic areas increased in a statistically significant manner (Fig. 7), while CD-31 reported blood vessel density slightly increased in a non-statistically significant manner (Fig. 8). This could be contributed to the following confounding factors: (1) during image acquisition, we may have selected a focal region of interest with aberrant vessel density (as shown in a representative image in Fig. 8A) and (2) low sensitivity of anti-CD31 staining with respect to new vessel development. CA-IX and CD31 are considered the gold standards when using immunohistochemical techniques to study tissue hypoxia and vessel formation, which allows us to examine the endpoint changes in hypoxia and vessel density [39]. However, it does not allow us to study longitudinal changes in oxygen saturation during and after the course of treatment. These markers may also not be sensitive enough to capture the small changes in hypoxia and vasculature during treatment [40].”This limitation has led many researchers to use optical techniques to non-invasively study the tumor microenvironment.

New optical techniques like diffuse reflectance spectroscopy (DRS) have been developed to study the tumor microenvironment over time. Other work has used spectroscopy techniques (near-infrared (NIR) spectroscopy) to study tumor optical properties like oxygen saturation and total hemoglobin, in addition to lipid and water content [41]. This work shows that during the course of treatment, the addition of a CCL2 blockade to 5-FU does slightly improve tissue-derived metrics when compared to a control vehicle. 5-FU alone showed improvement in oxygen saturation and oxyhemoglobin, while anti-CCL2 only showed no significant improvements in any of the DRS metrics. For the last result section (comparisons between IHC and DRS), we wanted to use a correlation plot to determine if our DRS modality is comparable to the golden standard of IHC work. Based on our results, we observed that there is a correlation between the CA-IX hypoxic calculations and the oxygen saturation that is obtained from DRS, which could indicate that DRS could be used to monitor tumor oxygen saturation during treatment. For vessel density, we observed no clear correlation between vessel density and oxygen saturation. Future work would include creating a new optical modality that can image blood vessels to determine vessel density and be a better predictor of tumor oxygenation. Overall, our results indicate that DRS may be a useful tool in monitoring early-tumor therapeutic responses in CRC. However, due to limited penetration depth, a fixed study area, and the addition of the skin layer, the accuracy of these metrics is limited since the whole tumor could not be measured. Additionally, single-modal DRS may be limited in providing clinicians relevant information which suggests that DRS should be combined with other endoscopically compatible imaging or spectroscopic methods.

The findings in this work suggest that the blockade of CCL2 is sufficient in the reduction of TAMs that are recruited into the tumor microenvironment and has the ability to modestly alter tumor perfusion during early-tumor response to treatment even though the overall benefit is relatively modest. These findings also suggest that DRS can be a valid tool to monitor physiological changes in a subcutaneous tumor during and even after a course of treatment. Even though our results did indicate some statistical differences in the DRS and IHC data, the utility of this approach may be limited for longitudinal monitoring of a subcutaneous tumor due to the tumor volume is larger than that of the optical probe used. Future studies using a chemically-induced orthotopic model of colon cancer using anti-CCL2 in combination with 5-FU while using DRS to monitor treatment response can provide clinicians more clinically relevant evidence of using cytokine-targeted therapy in CRC.

## Supplementary Information

**Additional file 1:** Longitudinal comparisons of DRS-derived oxyhemoglobin and deoxyhemoglobin.

## Data Availability

The authors declare that all data supporting the findings of this study are available within the article and its supplementary information files. A public repository of our datasets can be found in this FigShare link: https://figshare.com/projects/TAM_Study_-_Raw_Files/131684

## Abbreviations

5-FU: 5-Fluorouracil
CA-IX: Carbonic anhydrase-IX
CCL2: Macrophage chemoattractant protein-1
CRC: Colorectal cancer
CRT: Chemoradiotherapy
DRS: Diffuse reflectance spectroscopy
GF: Growth factors
IHC: Immunohistochemistry
MMPs: Matrix metalloproteases
pCR: Pathologic complete response
SDS: Source detection separations
StO_2_: Tissue oxygen saturation
TAMs: Tumor associated macrophages
THC: Tissue hemoglobin content
TME: Total mesorectal excision surgery
VEGF: Vascular endothelial growth factor
