# Supplementary material for: Macrophage-targeted anti-CCL2 immunotherapy enhances tumor sensitivity to 5-fluorouracil in a Balb/c-CT26 murine colon carcinoma model measured using diffuse reflectance spectroscopy
Source: BMC Immunol. 2022 Apr 23;23:20. doi: 10.1186/s12865-022-00493-5 (PMC9035255; doi:10.1186/s12865-022-00493-5)
Supplement: Supplementary file 1 — Additional file 1: Longitudinal comparisons of DRS-derived oxyhemoglobin and deoxyhemoglobin. [file 12865_2022_493_MOESM1_ESM.docx]

**Supplemental Information**

**Macrophage-targeted anti-CCL2 immunotherapy enhances tumor sensitivity to 5-fluorouracil in a *Balb/*c-CT26 murine colon carcinoma model measured using diffuse reflectance spectroscopy**

**Shelby N. Bess^1^, Gage J. Greening^1^, Narasimhan Rajaram^1^, Timothy J. Muldoon^1^***

**^1^Department of Biomedical Engineering, University of Arkansas, 1 University of Arkansas, Fayetteville, AR 72701**

*** Corresponding author**

**Email: tmuldoon@uark.edu**

**Results**

*DRS-derived metrics quantify tumor response during treatment*

*
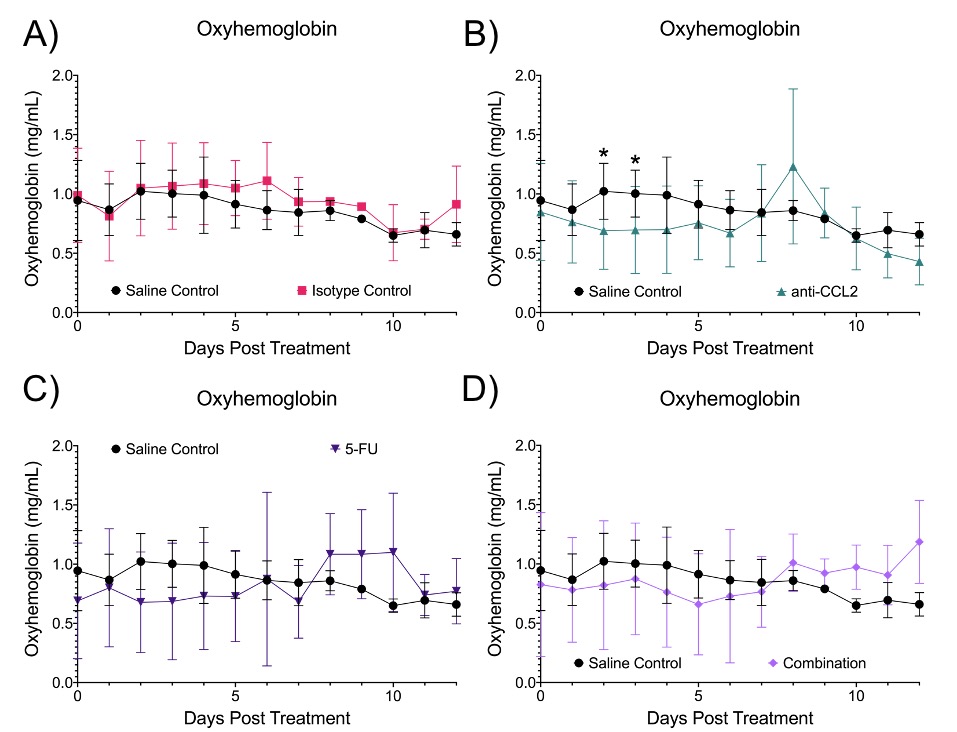
*

**Supplementary Figure 1. Longitudinal comparisons in oxyhemoglobin** Each panel shows the comparison of oxygen saturation in comparison to the saline and isotype control. A) Isotype control B) anti-CCL2 C) 5-FU and D) Combination. Plots created in Prism (GraphPad).

*
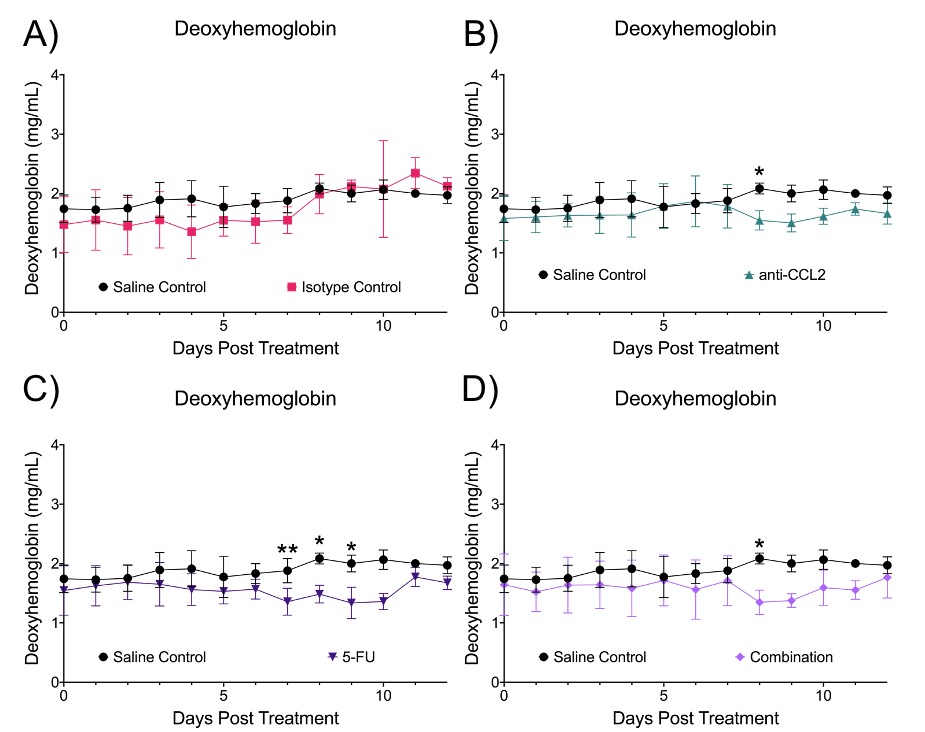
*

**Supplementary Figure 2. Longitudinal comparisons in deoxyhemoglobin** Each panel shows the comparison of oxygen saturation in comparison to the saline and isotype control. A) Isotype control B) anti-CCL2 C) 5-FU and D) Combination (* p ≤ 0.05). Plots created in Prism (GraphPad).
